# Supplementary material for: RUNX proteins desensitize multiple myeloma to lenalidomide via protecting IKZFs from degradation
Source: Leukemia. 2019 Feb 13;33(8):2006–21. doi: 10.1038/s41375-019-0403-2 (PMC6687534; doi:10.1038/s41375-019-0403-2)
Supplement: Supplementary file 1 — Supplementary Figure Legend [file 41375_2019_403_MOESM1_ESM.docx]

**Supplementary Figure Legends**

**Supplementary Figure 1. Dimerization independent association of IKZF1 to RUNX1.**

**(a)** The cell extracts of ARP-1 cells transfected with siRNAs targeting the indicated RUNX and IKZF proteins were probed with antibodies to the indicated proteins.

**(b)** HEK293T cells were transfected with FLAG-IKZF1 and increasing amounts of un-tagged RUNX1. FLAG-immunoprecipitates were probed with antibodies to the indicated proteins.

**(c)** As in *(b)* except that FLAG-IKZF1(1-400) truncation mutant was utilized.

**(d)** View of the sequence homology between the ZnF (1-4) in IKZF1 and IKZF3

Unless otherwise noted, immunoblots are representative of three independent experiments.

**Supplementary Figure 2. RUNX proteins desensitize multiple myeloma cells to the effects of lenalidomide.**

**(a)** Calculation of EC_50_ and EC_80_ based on lenalidomide induced degradation of IKZF1 (left) and IKZF3 (right) in OPM-1 cells under the indicated conditions are shown.

**(b)** The cell extracts of the indicated multiple myeloma cell lines were probed with antibodies to the indicated proteins.

**(c)** Left, cell counts of ARP-1 and RPMI-8226 cells for the indicated time points (mean±s.d., n=3 independent experiments). Right, the cell extracts of ARP-1 and RPMI-8226 cells expressing the indicated gRNAs were probed with antibodies to the indicated proteins.

**(d)** Cell counts of OPM-1 cells treated with DMSO or the indicated doses of lenalidomide. Every 3 days, cells were counted and re-plated in fresh media (mean±s.d., n=3 independent measurements, two-way ANOVA compared to DMSO, ***P* value≤0.01, *****P*≤0.0001).

**(e)** Same as in *(d)* except that pomalidomide was used (mean±s.d., n=3 independent measurements, two-way ANOVA compared to DMSO, **P* value≤0.05, ***P* value≤0.01, *****P*≤0.0001).

**(f)** Same as in *(d)* except that bortezomib was used on the indicated OPM-1 cells (mean±s.d., n=3 independent measurements).

Unless otherwise noted, immunoblots are representative of three independent experiments.

**Supplementary Figure 3. Effect of RUNX inhibitor on IKZFs binding.**

**(a)** Purified GST-tagged proteins as indicated were incubated with *in vitro* translated IKZF1 (left) or IKZF3 (right) and incubated with the RUNX inhibitor (AI-10-104) at indicated concentrations. GST pull-downs were probed with anti-IKZF1 and-IKZF3 antibodies, as indicated. Ponceau S staining shows the expressions of GST-proteins. The blue asterisk indicates GST-IKZF1 and GST-IKZF3.

**(b)** Levels of *IRF4* and *MYC* mRNA were analyzed by qRT-PCR under the indicated conditions. Lenalidomide and AI-10-104 were used at 1μM for 48 and 60 hours, respectively. (mean±s.d., n=3 independent measurements, one-way ANOVA compared to DMSO, **P*≤0.05, ***P*≤0.01, *****P*≤0.0001).

**Supplementary Figure 4. RUNX1 over-expression protects IKZFs from lenalidomide-induced degradation and toxicity.**

**(a)** Immunoblot analysis of whole cell lysates from NCI-H929 cells overexpressing RUNX1 or an empty vector (E.V.) treated with lenalidomide for 36 hours at the indicated concentrations.

**(b)** Calculation of EC_50_ and EC_80_ based on lenalidomide induced degradation of IKZF1 (left) and IKZF3 (right) in NCI-H929 cells under the conditions shown in *(a).*

**(c)** Cell counts of NCI-H929 cells overexpressing RUNX1 or empty vector (E.V.) grown in media containing DMSO or 1 μM lenalidomide (mean±s.d., n=3 independent experiments, two-way ANOVA, *****P*≤0.0001).

**(d)** Immunoblot analysis of whole cell lysates from OPM-1 cells treated with DMSO or 1 μM AI-10-104 for 36 hours. Following, lenalidomide was added for 24 hours at the indicated concentrations.

**(e)** Calculation of EC_50_ and EC_80_ based on lenalidomide induced degradation of IKZF1 and IKZF3 in OPM-1 cells under the indicated conditions are shown.

Unless otherwise noted, immunoblots are representative of three independent experiments.

**Supplementary Figure 5. RUNX inhibition potentiates the transcriptional response induced by lenalidomide.**

Volcano plot showing up- and down-regulated mRNAs in OPM-1 cells treated with 0.1 μM lenalidomide (LEN), 1 μM RUNX inhibitor (AI-10-104) or combination for 48 hours and compared to DMSO (n=3 independent experiments, DeSeq2).

**Supplementary Figure 6. Schematic model of lenalidomide and AI-10-104 combinatorial therapy in multiple myeloma**

RUNX proteins can compete with CRBN for interaction with IKZFs, thus protecting IKZFs from lenalidomide-induced degradation. Low doses of lenalidomide don’t display inhibitory effect on MM cell proliferation, however combinatorial treatment of cells with a RUNX inhibitor (AI-10-104) and lenalidomide inhibits cell viability. Mechanistically, AI-10-104 induces RUNXs dissociation from IKZF1 and IKZF3 so that CRBN can interact with IKZFs and promote IKZFs degradation.
